# Exposure to Particulate Matter in the Broiler House Causes Dyslipidemia and Exacerbates It by Damaging Lung Tissue in Broilers

**DOI:** 10.3390/metabo13030363

**Published:** 2023-02-28

**Authors:** Dan Shen, Qi Guo, Kai Huang, Weijia Mao, Kai Wang, Wenjie Zeng, Yansen Li, Zhendong Guo, Kentaro Nagaoka, Chunmei Li

**Affiliations:** 1 Research Centre for Livestock Environmental Control and Smart Production, College of Animal Science and Technology, Nanjing Agricultural University, Nanjing 210095, China; 2 Military Veterinary Research Institute, Academy of Military Medical Sciences, Changchun 130117, China; 3 Laboratory of Veterinary Physiology, Department of Veterinary Medicine, Faculty of Agriculture, Tokyo University of Agriculture and Technology, Tokyo 183-8509, Japan

**Keywords:** particulate matter, lung injury, inflammatory response, dyslipidemia, broiler house

## Abstract

The high concentration of particulate matter (PM) in broiler houses seriously endangers the biological safety of broilers and causes low growth performance, deserving more attention. This study aimed to investigate the effects of PM collected from a broiler house on the lung and systemic inflammatory responses and liver lipid anabolic process in broilers. Broilers were systemically exposed to fresh air (control) and 4 mg·m^−3^ and 8 mg·m^−3^ total suspended particles (TSP). Lung, liver, and serum were sampled after 7 (E7) and 14 (E14) days of PM exposure and 7 days after self-recovery (R 7). Corresponding kits were used to assay the inflammatory cytokines and serum biochemical indicators. The expression levels of genes related to lipid metabolism were detected by real-time polymerase chain reaction (RT-PCR) assay. The results showed a significant decrease in the average daily gain in broilers for 7 days of PM exposure (*p* < 0.05) and clear lung and liver inflammations in PM groups. In addition, upregulation of lung interleukin (IL)-1β and IL-8 and serum low-density lipoprotein cholesterol (LDL-C) and triglyceride (TG) occurred after 7 days of PM exposure (*p* < 0.05), and upregulation of lung serum tumor necrosis factor (TNF)-α and cholesterol (CHOL) occurred after 14 days of PM exposure (*p* < 0.05). A decrease in serum total antioxidant capacity (T-AOC) and glutathione peroxidase (GSH-px) levels was found after 14 days of PM exposure (*p* < 0.05), and the GSH-px level was maintained until 7 days after cessation of exposure (*p* < 0.05). Seven days after cessation of exposure, the expression levels of 3-hydroxy-3-methylglutaryl-CoA synthase 2 (*Hmgcs2*) and fatty acid synthase (*Fas*) genes significantly increased (*p* < 0.05) and decreased (*p* < 0.05), respectively. These results demonstrate that exposure to PM in broiler houses can induce systemic inflammation and dyslipidemia through local pulmonary inflammation and also exert toxic effects on the liver by disturbing the expression of genes involved in the hepatic lipid anabolic process.

## 1. Introduction

Severe indoor air pollution problems in livestock houses emerge with the high-density and intensive development of livestock production, and respiratory problems follow. The high concentrations of pollutants in farms increase the health burden of workers and animals and harm the surrounding atmospheric environment. One of the major environmental pollutants, particulate matter (PM), has contributed significantly to respiratory diseases. The hazard of fine particulate matter (PM_2.5_, aerodynamic diameter ≤ 2.5 µm) deserves special attention because it can penetrate through the airway and enter the pulmonary alveoli based on its small size [1]. Agricultural workers in farms with high concentrations of PM are more susceptible to respiratory diseases, such as pneumonia [2], asthma [3], and chronic obstructive pulmonary disease (COPD) [4]. Even residents who live in the neighborhood are at a higher risk of these diseases [5]. Animals kept in such houses are more likely to suffer from poor welfare and respiratory diseases, which further induce other secondary diseases, resulting in low productivity [6]. Poultry are more susceptible to respiratory diseases than mammals because of their unique respiratory tract structure [7]. The primary bronchi run through the entire lung tissue, and the tertiary bronchi form circulatory channels, making it easy for the airborne PM to spread in the lung. In addition, the air sac structure is unique to poultry, and PM tends to accumulate in the nine air sacs with a semi-open structure, causing air sacculitis.

In addition to the unique respiratory tract structure of poultry, the concentrations of PM are also higher in poultry houses than in pig or cattle houses and higher in broiler houses with litter systems than in hen houses with cage systems [6]. The PM concentrations in broiler houses are exceptionally high in winter due to insufficient ventilation. They also vary with other factors such as variety, age, and feeding behavior. They can reach peak values of 6.5 mg·m^−3^ and 81.33 mg·m^−3^ for PM_2.5_ and PM_10_, respectively [8]. The concentrations of PM_10_ (aerodynamic diameter ≤ 10 µm) and total suspended particles (TSP, PM with aerodynamic diameter ≤ 100 µm) in poultry houses were limited to 4 mg·m^−3^ and 8 mg·m^−3^, respectively, by the recommended standards for agricultural industry (NT/T) 388–1999 promulgated by Ministry of Agriculture of the People’s Republic of China [9] for the sake of the health and welfare of farmworkers and poultry. Moreover, the composition of PM from broiler houses is complex. Organic matter contributes most to PM in poultry houses rather than combustion materials [10], which is different from atmospheric particles [11]. PM_2.5_ in poultry houses is mainly derived from manure, feed, bedding materials, and feathers [10,12]. Thus, there are many detrimental substances adhered to its surfaces. Verifiably, it was detected to contain a large amount of organic carbon, heavy metals, ions [10], bacteria, fungi, endotoxins [13], and allergens [12,14], indicating its potential hazard to respiratory health.

The lung is the primary organ targeted by airborne PM_2.5_. The PM_2.5_ of poultry houses with complex composition enters the alveoli and stimulates macrophages to release pro-inflammatory factors. These factors recruit and stimulate other immune cells, alveolar epithelial cells, endothelial cells, and fiber cells to secrete many cytokines and adhesion factors, forming an inflammatory cascade reaction [15]. In addition, it can also cause oxidative stress through various pathways [16] and induce apoptosis [17], pyroptosis [18], or autophagy [19]. The alveolar surfactant is also destroyed [20], resulting in alveolar collapse and shrinkage, and then lung tissue damage is aggravated. Lung inflammation and injury are signals of various respiratory diseases. PM_2.5_ in poultry houses can considerably increase the incidence of respiratory diseases [21] and decrease the immune performance in broilers [22,23], leading to other secondary diseases.

Many studies have reported that PM_2.5_ exposure can induce distinct inflammatory signaling in the lung and liver [24]. Tumor necrosis factor (TNF)-α associated hepatic dyslipidemia [25] and cardiac hypertrophy [26] cause liver fibrosis [27,28] and elevate the risk of oxidative stress-driven nonalcoholic fatty liver disease [29]. These cases suggested that PM_2.5_ not only dwells in the lungs but also is likely to cross the air–blood barrier and adversely affect the liver through the blood circulation system. However, it is still unknown how PM_2.5_ in poultry houses will cause harm to the liver tissue after inducing lung tissue inflammation in broilers. Respiratory diseases and the secondary diseases induced by them seriously reduce the growth performance and survival rate of broilers [30], resulting in economic losses. To solve this problem, the harm caused by PM_2.5_ to broilers’ lung tissue, blood indices, and liver tissue should be clarified.

The traditional treatment methods commonly used in relevant studies are nasal or tracheal infusion. The grasping or anesthesia process will cause animal stress and affect physiological indicators. Therefore, the non-contact systemic exposure method was used in this study to explore the effects of PM in the poultry houses on lung tissue and blood indices, liver function, and expression of genes related to liver lipid metabolism in Arbor Acres (AA) broilers. This study will provide a theoretical basis for effectively preventing and treating respiratory diseases and liver damage in broilers caused by particulate matter in broiler houses.

## 2. Materials and Methods

### 2.1. PM Collection

The PM was collected from a commercial broiler house located in Yangzhou city, Jiangsu province, China (east longitude 119°01′ to 119°54′, north latitude 32°15′ to 33°25′), for the subsequent exposure experiment, which maximized approaching the composition of PM inhaled by broilers in the actual breeding process. The commercial broiler house, a standard mode of broiler breeding, is a cage-free system with dimensions of 90 × 11 m and is bedded with rice hull. It is equipped with a longitudinal negative pressure ventilation system, and about 15,000 mixed-sex broilers aged 21–42 days old were housed inside during the collection period. A TSP sampler (Laoying Incorporated, Qingdao, China) with a flow rate of 100 L·min^−1^ was used to collect TSP at the height of 1.0 m in the middle of the house for 23 h per day. Then, the collected PM was sealed in a sterile plastic bag and stored at 4 °C. After collection, all PM samples were thoroughly mixed and immediately used for the next exposure experiment.

### 2.2. Animals

Seventy-two 14-day-old AA broilers were purchased from an intensive commercial broiler farm. They were then raised in the Animal Husbandry Laboratory. The temperature and relative humidity in the animal house were controlled at 20–24 °C and 55%, respectively, with a 16 h light and 8 h dark cycle. The broilers were fasted and water-free during the exposure time and had free access to feed and water at other times of the experiment.

Animal care and use were implemented with the guidance of the Animal Ethics Committee of Nanjing Agricultural University.

### 2.3. Experimental Design

All broilers were preliminarily fed for 1 week before the formal trial to adapt to the new environment. A total of 72 21-day-old broilers were randomly divided into 3 groups, and 24 healthy broilers (half male and half female) of similar weight (0.52 ± 0.01 kg) were in each group. In the control group, broilers were exposed to fresh air in the exposure room with TSP concentration of 0.657 ± 0.117 mg·m^−3^. In the low PM group, broilers were exposed to TSP concentration of 4 mg·m^−3^. It is the actual airborne TSP concentration measured in a commercial broiler house equipped with rice husk bedding, where the PM is collected from. The PM concentrations of different sizes in the house were measured at 15 uniformly distributed points from 7:00–19:00 every 2 h for a 3-d monitoring period by a DustTrak II model 8533 aerosol monitor (TSI Incorporated, Shoreview, MN, USA) with detection ranging from 0.001 to 150 mg·m^−3^ and measurement accuracy of ± 0.001 mg·m^−3^. In the high PM group, broilers were exposed to a TSP concentration of 8 mg·m^−3^, per the limited value of TSP concentration in poultry houses stipulated by the NT/T 388–1999 Ministry of Agriculture of the People’s Republic of China [9].

### 2.4. Exposure Treatment

Different from the nasal drip, tracheal drip, and other traditional contact treatment methods, a systemic exposure method was chosen to replicate the natural state of broiler exposure to PM in a commercial farm and avoid the stress caused by the treatment method itself. The exposure treatments were conducted in three identical adjacent exposure chambers to keep all environmental factors consistent except PM concentrations. Broilers were raised in cages and placed in a rearing room with a TSP concentration of 1.198 ± 0.077 mg·m^−3^ during the non-exposure period. They were transferred to separate identical chambers with a size of 1.0 × 0.8 × 0.5 m in an exposure room with a TSP concentration of 0.126 ± 0.002 mg·m^−3^ before exposure treatment. A hollow plastic hose connected one end to the exposure chamber and the other to a BT901 model dry dispersive injection system (Dandong Baite Incorporated, Dandong, China). The PM was added into the injection port of the instrument and sprayed into the chambers through the hose under pump pressure, and the computer terminal controlled the injection speed. The real-time PM concentrations of different sizes in the exposure chambers were measured and recorded by DustTrak Ⅱ model 8533 aerosol monitors (TSI Incorporated, Shoreview, MN, USA) during the whole exposure period. They are shown in Supplementary Table S1 and Figure S1, and the amount of PM added was adjusted accordingly to control the PM concentrations inside chambers. Broilers in two PM groups were exposed to PM for 2 h a day from 8:30 to 10:30 for 14 days, and simultaneously, the others in the control group were exposed to indoor fresh air without the addition of any PM. After daily exposure, they were all transferred back to cages in the rearing room. The three exposure chambers were cleaned with water and then irradiated with ultraviolet lights for sterilization for 16 h until the subsequent exposure treatment. The used monitors were rotated in the three chambers to eliminate measurement errors.

### 2.5. Sample Collection

Feed consumption and weight of broilers in each group were measured and recorded weekly from 21 to 42 days of age. Seven days after exposure to PM or indoor air, 8 broilers were randomly selected from each group for sacrifice and sampling of the blood and viscera of the heart, liver, spleen, lung, and bursa of Fabricius. Serum was extracted after static settlement and centrifugation (3000 r·min^−1^, 10 min, 4 °C) for the determination of serum biochemical indices. The viscera were weighed and then the organ index was calculated. A portion of tissue (approximately 0.5 × 0.5 × 0.5 cm) was taken from the middle part of each broiler’s right lung and liver and then was fixed in 4% paraformaldehyde solution. The samples of tissues were harvested as uniformly located as possible. Parts of each broiler’s left lung and liver were placed in labeled freezing-storage tubes after being cut up and thoroughly mixed. Samples were then stored at −80 °C until the determination of lung inflammatory factors and liver lipid metabolism gene expression levels. The same sample collection and treatment were performed after 14 days of PM exposure and 7 days of self-recovery.

### 2.6. Histomorphology Examination

The lung and liver tissue were fixed in 4% paraformaldehyde solution for 24 h and then dehydrated, clarified, wax-penetrated, and paraffin-embedded into tissue blocks. Sections with a thickness of 5 μm were obtained by serial sectioning. Hematoxylin and eosin (H&E) staining was performed to observe and evaluate lung and liver damage.

### 2.7. Inflammatory Cytokines and Serum Biochemical Assay

The following inflammatory cytokines in supernatant of lung homogenate (3000 rpm, 20 min, 4 °C) and serum were determined: tumor necrosis factor α (TNF-α), interleukin (IL)-1β, IL-6, IL-8, IL-10. According to the corresponding manufacturer’s instructions, ELISA kits were used detect them with the competition method. The serum biochemical indices that related to antioxidant enzyme and liver function were assayed, including total antioxidant capacity (T-AOC), total superoxide dismutase (T-SOD), catalase (CAT), glutathione peroxidase (GSH-px), albumin, alkaline phosphatase (AKP), alanine aminotransferase (ALT), aspartate aminotransferase (AST), and total bilirubin (T-BIL). All these test kits were purchased from Nanjing Jiancheng Bioengineering Institute (Nanjing, China). Serum lipid-related biochemical indicators, total protein (TP), low-density lipoprotein cholesterol (LDL-C), glucose (GLU), cholesterol (CHOL), and triglyceride (TG), were detected with test kits (Meikang Incorporated, Ningbo China) using a 7020 Automatic Analyzer (Hitachi Incorporated, Tokyo, Japan).

### 2.8. RT-PCR Assay

Total ribonucleic acid (RNA) of the liver was extracted using a solution of Trizol (Vazyme Incorporated, Nanjing China) and then reverse-transcribed to complementary deoxyribonucleic acid (cDNA) using Goldenstar RT6 cDNA Synthesis Kit Ver.2 (Tsingke Incorporated, Beijing, China) according to corresponding instructions. The cDNA samples were diluted to 500 ng·μL^−1^ with double distilled water (ddH_2_O) after detecting their concentration and quality with a value of OD260/280 using a NanoDrop 2000 spectrophotometer (Thermo Fisher Scientific Incorporated, Massachusetts, USA). Quantitative real-time polymerase chain reaction (qRT-PCR) was performed with 2 × T5 Fast qPCR Mix (SYBR Green Ⅰ) kit (Tsingke Incorporated, Beijing, China) using a QuantStudio5 Real-Time PCR Instrument (ABI Incorporated, San Ramon, CA, USA). The qRT-PCR reactions were conducted in 20 μL of reagent containing 2 μL cDNA, 0.8 μL each primer (10 μM), 10 μL 2 × T5 Fast qPCR Mix (SYBR Green Ⅰ), 0.4 μL 50 × Rox Reference Dye Ⅱ, and 6.0 μL ddH_2_O. The expression levels of the genes *Fas* (fatty acid synthase), *Hmgcr* (3-hydroxy-3-methylglutaryl-CoA reductase), *Hmgcs2* (3-hydroxy-3-methylglutaryl-CoA synthase 2), and *PPARα* (peroxisome proliferator-activated receptor alpha) were determined by the method mentioned above. Target genes’ relative messenger RNA (mRNA) expression levels were normalized to β-actin expression and calculated with the 2^−ΔΔCt^ method [31]. The primer sequences of genes were obtained from the National Center of Biotechnology Information (NCBI) and synthesized by Shanghai Sangon Biotech Co., Ltd. (Supplementary Table S2).

### 2.9. Statistical Analysis

Data analysis was performed using GraphPad Prism Version 8.0 software. One-way analysis of variance (ANOVA) followed by Tukey’s multiple comparisons was used for statistical analysis. Values were expressed as the mean ± standard error of the mean (SEM), and *p* < 0.05 was considered a statistically significant difference.

## 3. Results

### 3.1. Growth Performance

As shown in Table 1, average daily gain significantly decreased when broilers were exposed to a PM concentration of 8 mg·m^−3^ for 7 days (*p* < 0.01). This significant difference disappeared after 14 consecutive days of exposure (*p* > 0.05), and the tendency was similar 7 days after recovery.

No significant difference was observed in broilers’ organ weight and organ index after PM exposure for 7 days or 14 days (*p* > 0.05, Table S3).

### 3.2. Lung and Liver Histomorphology

**Figure 1:**
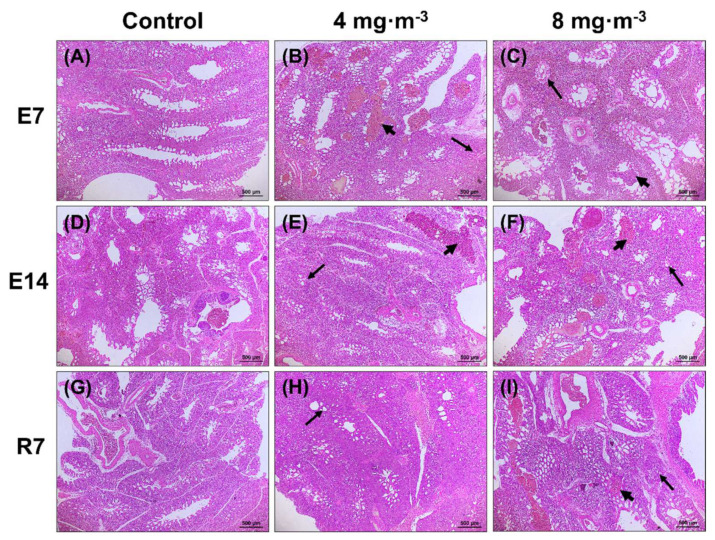
Effects of particulate matter exposure on lung histomorphology of broilers. Hematoxylin and eosin (H&E) staining of the lung. (**A**,**D**,**G**) represent the control group after E7, E14, and R7, respectively; (**B**,**E**,**H**) represent the exposure group at PM concentration of 4 mg·m^−3^ after E7, E14, and R7, respectively; (**C**,**F**,**I**) represent the exposure group at PM concentration of 8 mg·m^−3^ after E7, E14, and R7, respectively. The stubby arrows show hemorrhaging in the lungs and bronchi filled with blood cells. The elongated arrows indicate pulmonary capillary shrinkage collapse and deformation. E7, exposure for 7 days; E14, exposure for 14 days; R7, recovery for 7 days.

As shown in Figure 1, compared with the control group, both low and high PM concentrations at 7 and 14 days of exposure can cause lung hemorrhage; blood cells entered the bronchi of broilers, and pulmonary capillary constriction was also present. After recovery, 7 days after cessation of PM exposure, the symptoms of lung congestion were relieved, but there were still lesions in the high PM concentration group.

**Figure 2:**
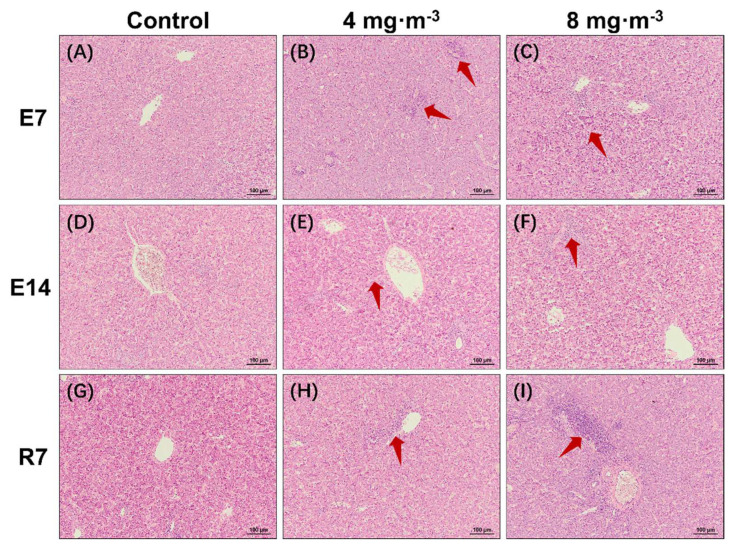
Effects of particulate matter exposure on liver histomorphology of broilers. Hematoxylin and eosin (H&E) staining of the liver. (**A**,**D**,**G**) represent the control group after E7, E14, and R7, respectively; (**B**,**E**,**H**) represent the exposure group at PM concentration of 4 mg·m^−3^ after E7, E14, and R7, respectively; (**C**,**F**,**I**) represent the exposure group at PM concentration of 8 mg·m^−3^ after E7, E14, and R7, respectively. Arrows indicate inflammatory cell infiltration. E7, exposure for 7 days; E14, exposure for 14 days; R7, recovery for 7 days.

For the liver, suspected inflammatory cell infiltration was observed in the liver tissue of PM-exposed broilers (Figure 2).

### 3.3. Inflammatory Factor Levels

**Figure 3:**
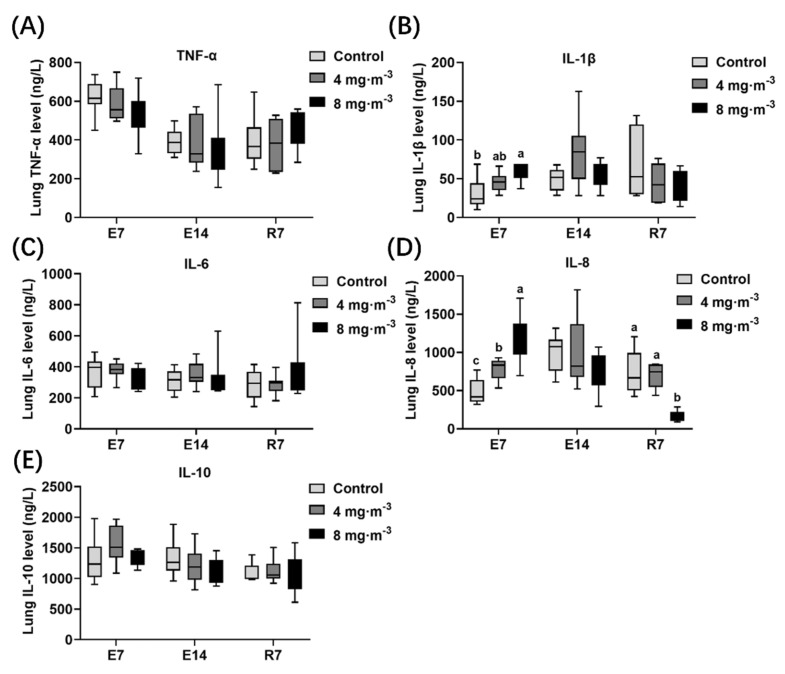
Effects of particulate matter exposure on inflammatory factor levels in lung of broilers. (**A**–**E**) represent the TNF-α, IL-1β, IL-6, IL-8, and IL-10 levels in lung tissue of broilers after E7, E14, and R7, respectively. Data are presented as mean ± SEM of groups (n = 8). Different letters indicate statistically significant differences between groups (*p* < 0.05). E7, exposure for 7 days; E14, exposure for 14 days; R7, recovery for 7 days.

The inflammatory factor levels in the lungs of broilers in control and PM-exposed groups were detected (Figure 3). The lung IL-1β (Figure 3B) level in broilers of 8 mg·m^−3^ group significantly increased after 7 days of PM exposure compared to the control group (*p* < 0.05). Furthermore, IL-8 (Figure 3D) levels were significantly increased in two PM-exposed groups in comparison with the control group when they were exposed to PM for 7 days (*p* < 0.05). Interestingly, the difference disappeared after 14 days of PM exposure. Moreover, the IL-8 level in the 8 mg·m^−3^ group decreased remarkably compared to the control and 4 mg·m^−3^ groups after 7 days of recovery (*p* < 0.05).

**Figure 4:**
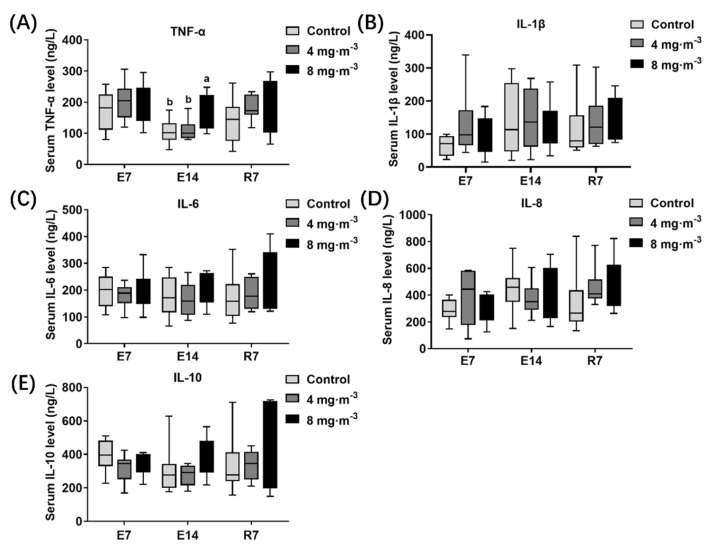
Effects of particulate matter exposure on inflammatory factor levels in serum of broilers. (**A**–**E**) represent the TNF-α, IL-1β, IL-6, IL-8, and IL-10 levels in serum of broilers after E7, E14, and R7, respectively. Data are presented as mean ± SEM of groups (n = 8). Different letters indicate statistically significant differences between groups (*p* < 0.05). E7, exposure for 7 days; E14, exposure for 14 days; R7, recovery for 7 days.

The inflammatory factor levels in the serum of broilers in each group are shown in Figure 4. No significant difference was observed in serum inflammatory factors detected in broilers after PM exposure for 7 days. The TNF-α (Figure 4A) level in broilers exposed to 8 mg·m^−3^ for 14 days was significantly increased compared to the control group (*p* < 0.05).

### 3.4. Antioxidant Capacity

**Figure 5:**
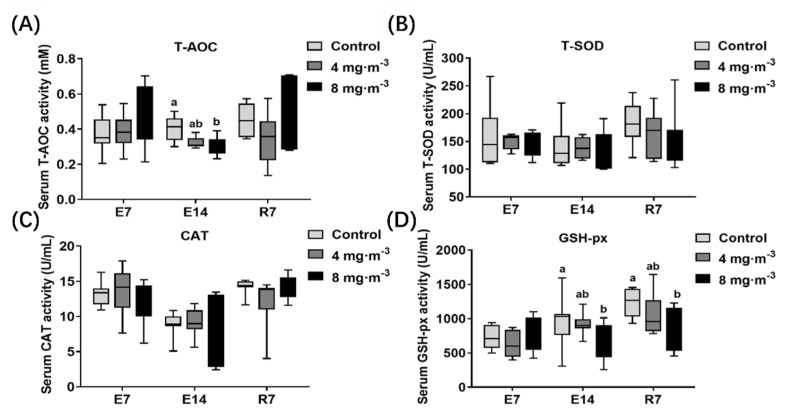
Effects of particulate matter exposure on the antioxidant enzyme content in serum of broilers. (**A**–**D**) represent the T-AOC, T-SOD, CAT, and GSH-px content in serum of broilers after E7, E14, and R7, respectively. Data are presented as mean ± SEM of groups (n = 8). Different letters indicate statistically significant differences between groups (*p* < 0.05). E7, exposure for 7 days; E14, exposure for 14 days; R7, recovery for 7 days.

As shown in Figure 5, no significant difference in any serum antioxidant index was detected in the treatment groups after 7 days of PM exposure compared to the control group (*p* > 0.05). The T-AOC (Figure 5A) and GSH-px (Figure 5D) content in the 8 mg·m^−3^ group were significantly decreased after 14 days of PM exposure compared to the control group (*p* < 0.05). Moreover, this significant difference in GSH-px persisted up to 7 days after cessation of exposure (*p* < 0.05).

### 3.5. Liver Function

**Figure 6:**
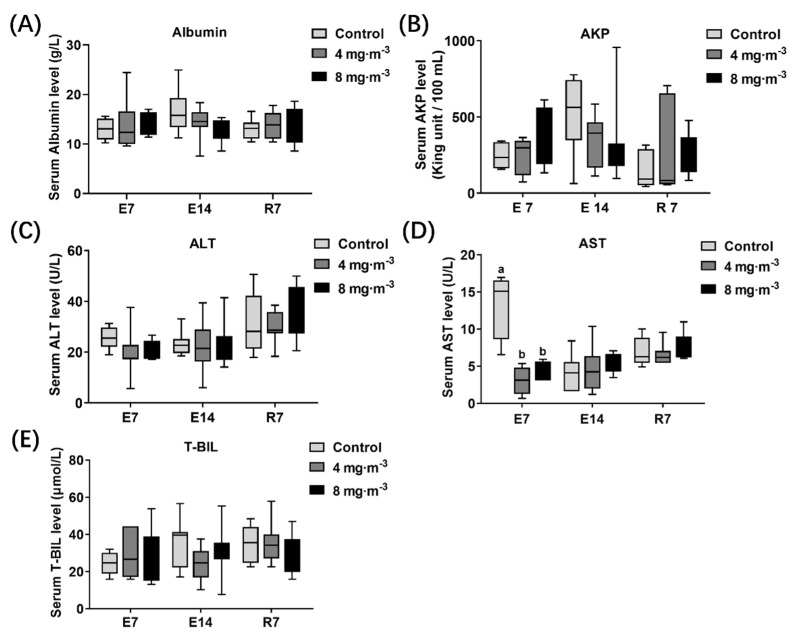
Effects of particulate matter exposure on serum biochemical indices related to liver function of broilers. (**A**–**E**) represent the albumin, AKP, ALT, AST, and T-BIL levels in serum of broilers after E7, E14, and R7, respectively. Data are presented as mean ± SEM of groups (n = 8). Different letters indicate statistically significant differences between groups (*p* < 0.05). E7, exposure for 7 days; E14, exposure for 14 days; R7, recovery for 7 days.

The serum biochemical indices related to liver function were detected in broilers (Figure 6). No significant difference was observed in albumin (Figure 6A), AKP (Figure 6B), ALT (Figure 6C), and T-BIL (Figure 6E) in serum among PM-exposed groups and the control group after 7 or 14 days of PM exposure (*p* > 0.05). The AST (Figure 6D) level decreased notably in both PM-exposed groups after 7 days of PM exposure compared to the control group (*p* < 0.05).

### 3.6. Lipid Concentration in Serum

**Figure 7:**
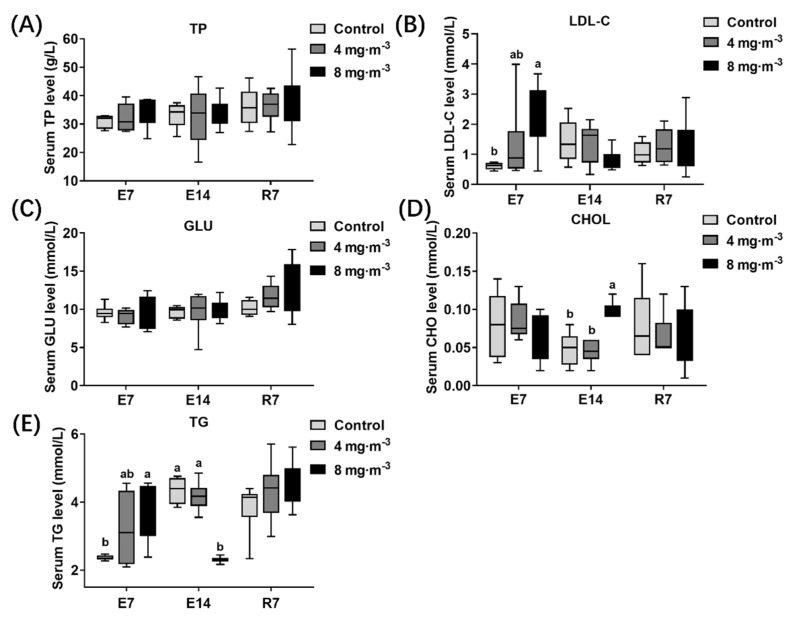
Effects of particulate matter exposure on lipid concentration in serum of broilers. (**A**–**E**) represent the TP, LDL-C, GLU, CHOL, and TG levels in serum of broilers after E7, E14, and R7, respectively. Data are presented as mean ± SEM of groups (n = 8). Different letters indicate statistically significant differences between groups (*p* < 0.05). E7, exposure for 7 days; E14, exposure for 14 days; R7, recovery for 7 days.

The three groups’ serum lipid concentrations in broilers were detected (Figure 7). Compared to the control group, the serum LDL-C (Figure 7B) and TG (Figure 7E) levels of broilers in the 8 mg·m^−3^ group were significantly increased after 7 days of PM exposure (*p* < 0.05), and the CHOL (Figure 7D) level was significantly increased after 14 days of exposure (*p* < 0.05). Notably, the TG level significantly declined when broilers were exposed to a PM concentration of 8 mg·m^−3^ for 14 days (*p* < 0.05).

### 3.7. Expression Levels of Genes Related to Lipid Metabolism in the Liver

**Figure 8:**
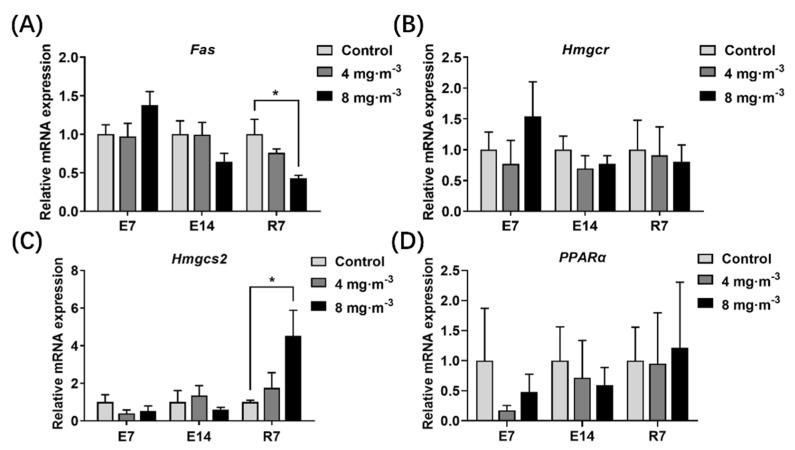
Effects of particulate matter exposure on relative mRNA expression levels of genes related to lipid metabolism in the liver of broilers. (**A**–**D**) represent the *Fas*, *Hmgcr*, *Hmgcs2*, and *PPARα* levels in serum of broilers after E7, E14, and R7, respectively. Data are presented as mean ± SEM of groups (n = 8). * *p* < 0.05. E7, exposure for 7 days; E14, exposure for 14 days; R7, recovery for 7 days.

The expression levels of genes related to lipid metabolism in the liver are shown in Figure 8. There were no remarkable changes in genes tested in broilers exposed to PM for 7 or 14 days (*p* > 0.05). Interestingly, compared to the control group, the *Fas* gene expression in the liver of broilers significantly declined after 7 days of recovery (*p* < 0.05, Figure 8A). The *Hmgcs2* gene expression significantly increased when broilers recovered for 7 days after PM exposure of 8 mg·m^−3^ for 14 days (*p* < 0.05, Figure 8C).

## 4. Discussion

### 4.1. Characteristics of PM during Broiler Rearing

The research focuses on atmospheric PM changed from TSP at the beginning to PM_10_ and finally to PM_2.5_, which can be deposited in the terminal bronchioles and alveoli through the respiratory tract, and even enter the blood circulation through the air–blood barrier, resulting in systemic adverse effects [32]. PM_2.5_ has been listed as high-risk inhaled particles by International Standardization Organization (ISO) 7708 [33]. The study of systemic exposure requires a large amount of PM, and the collection of PM_2.5_ is time-consuming and inefficient, making it unsuitable for the demands of this experiment. Although TSP has been collected and used for systemic exposure, it has been reported that coarse particulate matter (PM_2.5–10_, 2.5 µm ≤ aerodynamic diameter ≤ 10 µm) is usually blocked in the upper respiratory tract, and larger particles (aerodynamic diameter ≥ 10 µm) are kept in or out of the nasal cavity [32]. The respiratory tract of broilers is narrower than that of humans, and it is more difficult for coarse particles to enter the respiratory tract [34]. It has been reported that PM_5_ and PM_10_ could not be deposited in broilers’ alveoli and air sacs at 2 and 4 weeks due to large particle sizes, respectively. In addition, the deposition of PM_20_ to PM_1_ in the lung of broilers increased from 3% to 17% [35]. Thus, PM_2.5_ is also a significant contributor to the adverse effects in broiler houses.

Animal behaviors such as feeding, defecation, walking, and feather shedding are major sources of PM in animal houses [10,12], and the PM accumulates with the increase in animal age. That is, PM concentrations increase. The rearing and exposure rooms were both clean prior to the start of this experiment. All behaviors of experimental broilers took place in the rearing room during the non-exposure time and in the exposure chambers during exposure treatment. The exposure room was not contaminated throughout the growth cycle. That is why the TSP concentration of the rearing room was much higher than that in the exposure room, which reached 1.198 ± 0.077 mg·m^−3^. We detected the PM_2.5_ components in a broiler house. We found that it was attached with rich ions of SO_4_^2−^, NO_3_^−^, and NH_4_^+^; heavy metals of K and Fe; organic carbon (OC); pathogenic bacteria of *Staphylococcus*, *Corynebacterium*, and *Enterococcus*; harmful fungi of *Aspergillus*, *Scopulariopsis*, *Wallemia*, and *Fusarium*; abundant endotoxins; etc., indicating the harmfulness of PM_2.5_ in broiler houses [13].

### 4.2. Effects of PM Exposure on Productivity and Histomorphology in the Lung and Liver of Broilers

In order to clarify the effect of PM on the apparent health of broilers, we first paid attention to the changes in growth performance and lung and liver histomorphology after the different durations of PM exposure. The average daily gain of broilers was significantly decreased at 7 days of PM exposure, which was consistent with the results of previous studies [22]. However, this difference disappeared after 14 days of exposure and 7 days of self-recovery. This is in line with a report that the body weight of broilers significantly decreased after the first PM treatment but had no change after the second treatment [23]. This result may be explained by the fact that adaptive response and self-recovery occur in broilers, or the immune system is stimulated to play a protective role during long-term PM exposure. However, the immune system could be damaged irreversibly by excessive immune responses, allowing diverse pathologies to emerge [36].

The histomorphology also reflected that PM exposure could cause damage in broilers. Inflammatory infiltrates and hyperemia of the tertiary bronchus were observed in lung tissue, indicating that the air–blood barrier was destroyed. Then, serum macromolecules were allowed to enter the pulmonary capillary, and exogenous substances also were able to invade lung cells, triggering an inflammatory cascade by activating multiple signal transduction pathways [37]. In addition to lung tissue injury, we noticed that inflammatory cell infiltration occurred in the liver, indicating that the inflammatory response induced by PM_2.5_ is not limited to the respiratory tract but can also be detrimental to other organs. Furthermore, the lung and liver tissue injuries were dose-dependent but not time-dependent. The damage was more severe at 7 days than at 14 days of PM exposure, which is consistent with the tendency of growth performance. Subsequently, it was alleviated after discontinuation of exposure. However, it did not disappear, suggesting that PM-induced lung and liver injuries could not be fully recovered from or need a longer time to recover from.

### 4.3. Effects of PM Exposure on Inflammation Level and Antioxidant Capacity in Broilers

Various microbial-related molecular patterns on the surface of PM_2.5_ from poultry or pig houses can activate the TLRs/MAPK/NF-κB signaling pathway and the NLRP3 inflammasome in alveolar immune cells, including but not limited to alveolar macrophages and type Ⅱ epithelial cells. These immune cells release many inflammatory factors, such as TNF-α, IL-1β, IL-6, IL-8, IL-18, and cyclooxygenase (COX)-2, inducing apoptosis or cell rupture [38,39], and then the lung tissue injury is aggravated. That is why we found the occurrence of an inflammatory response after PM exposure. The levels of inflammatory factors, IL-1β and IL-8, in lung tissue increased significantly after 7 days of PM exposure. However, this difference disappeared after 14 days of exposure and cessation, which was consistent with the change tendency of growth performance and morphological lung damage mentioned above. A similar study concluded that the expression of immune-related proteins could be restored after short-term exposure to PM_2.5_ [40].

Noteworthily, the change in inflammation level in serum induced by PM exposure differs from that in lung tissue. There was no significant difference between the control and treatment groups after 7 days of PM exposure, while the TNF-α level significantly increased after 14 days of PM exposure, indicating systemic inflammation. TNF-α is a signaling molecule in the early stages of inflammation. It stimulates macrophages and epithelial cells to secrete IL-1β and IL-8, which recruit and activate neutrophils, exacerbating the local inflammatory response in the body [41]. According to the results, lung tissue is already in the middle stage of inflammation at 7 days of PM exposure, while systemic inflammation is not triggered yet. Then, the local inflammation in the lung tissue begins to shift to systemic inflammation after 14 days of PM exposure.

Similar to the trend of the serum inflammation factor level, serum antioxidant level decreased significantly at 14 days of PM exposure, as indicated by the indices of T-AOC and GSH-px, suggesting that long-term PM exposure reduced systemic antioxidant capacity [42]. The GSH-px level did not recover 7 days after cessation of exposure, indicating that the damage caused by PM was not completely reversible. Although in appearance, the growth performance of broilers recovered after long-term exposure to PM, the immune function and stress resistance of broilers were decreased, which may lead to increases in susceptibility to diseases under other stimulating conditions [36], such as increased NH_3_ concentrations or temperature shock inside the broiler houses.

### 4.4. Effects of PM Exposure on Liver Function and Lipid Metabolism in Broilers

It could be predicted that PM exposure will have detrimental effects on other organs through blood circulation based on the increased systemic inflammation and decreased antioxidant capacity mentioned above. Several studies have reported that PM_2.5_ can cause nonalcoholic fatty liver disease [29] and hepatic dyslipidemia [25]. This led us to question whether broilers exposed to high concentrations of PM experience abnormalities in liver function, lipid synthesis, and metabolism. To answer this question, we first measured liver function and lipid-related indices in serum. No statistically significant differences were shown in liver function indicators, while there was a notable increase in serum lipid content. The levels of TG and CHOL, two major components of blood lipids [43], increased significantly after 7 and 14 days of PM exposure, respectively, which is consistent with a previous report [25]. The CHOL transporter, LDL-C, which carries CHOL from the liver to other tissues through the blood and can cause hardening of the arteries at high concentrations [43], was also significantly increased at 7 days of PM exposure. An increase in the TG level followed by an abnormal and significant decrease after 14 days of exposure indicated dysfunction of hepatic lipid metabolism, which returned to a normal level 7 days after the cessation of PM exposure. These results indicated that exposure to PM in the broiler house increased broilers’ risk of dyslipidemia and cardiovascular disease.

The inflammatory response is critical in modulating the development of nonalcoholic fatty liver disease [44]. PM_2.5_ could accelerate hepatic inflammation by systemic inflammation through circulating inflammatory cytokines and direct stimulation of liver stellate macrophages [45]. Namely, there are two possible ways by which PM_2.5_ induces dyslipidemia. One is that PM_2.5_ enters the blood circulation due to its small particle size, stimulates the liver and fat cells to enhance lipid synthesis and release, and directly causes dyslipidemia. The other is that local inflammation in the lungs induced by PM_2.5_ spreads into the blood, stimulating the liver to synthesize lipids, thereby indirectly causing dyslipidemia. It can be confirmed that the former is one of the main mechanisms of PM_2.5_ in poultry house-caused dyslipidemia in broilers because it occurred at 7 days of PM exposure, which preceded the increase in serum inflammation level and the decrease in antioxidant level. Otherwise, it has also been reported that prolonged PM_2.5_ exposure induced dyslipidemia by triggering oxidative stress [29] and inflammation depending on the TNF-α pathway [25], which provides an explanation for the simultaneous increase in TNF-α and lipid concentrations and decrease in antioxidant capacity in this study. It was hypothesized that PM_2.5_ from the poultry house caused dyslipidemia in broilers by both the two patterns mentioned above. The former triggers dyslipidemia, while the latter drives its development.

The TG and CHOL accumulation in serum may be attributed to changes in genes regulating lipid metabolism in the liver. Therefore, we next analyzed the mRNA-level expression of genes involved in lipid anabolic metabolism in the liver. Unexpectedly, we found no significant change in hepatic lipid anabolic gene expression during PM exposure. However, a significant decrease in the expression of the *Fas* gene, encoding a critical rate-controlling enzyme in endogenous fatty acid synthesis [46], was detected 7 days after the cessation of PM exposure. This is disparate from the statement that lipid accumulation would induce *Fas* gene expression as reported in [47]. It may be a negative feedback effect after the elevation of blood lipid content in this experiment. On the contrary, the gene expression of *Hmgcs2*, encoding a key enzyme in ketone synthesis, increased significantly 7 days after cessation of PM exposure. The expression of *Hmgcs2* protein was upregulated to catalyze ketogenesis as compensation for hepatic lipid deposition [48]. Liver lipid accumulation occurs when the ketogenic response is disrupted, leading to nonalcoholic fatty liver disease [49]. Changes at the gene level may have occurred before the increase in serum lipid levels, that is, prior to 7 days of PM exposure, and thus were not shown at the time points detected in this study. After cessation of PM exposure, hyperlipidemia downregulated *Fas* gene expression and upregulated *Hmgcs2* expression with negative feedback decreasing the hyperlipidemia to the normal level. These results indicated that PM_2.5_ in poultry houses could cause hepatic dyslipidemia in broilers, which is also supported by published papers [26,29].

## 5. Conclusions

In conclusion, in terms of growth performance, PM exposure for 7 days reduced the average daily gain of broilers. Histologically, visible lung and liver injuries were observed in broilers exposed to PM. At the molecular level, PM exposure first induced local pulmonary inflammation and then systemic inflammation, accompanied by a decrease in systemic antioxidant levels. Further, PM with a small size entered the blood circulation, interfered with lipid synthesis and metabolic function in broiler livers at the genetic level, and in turn induced dyslipidemia. It is demonstrated that in addition to the damage to the respiratory tract of broilers, the PM in the broiler houses can also lead to apparent liver injury and dyslipidemia, which could be exacerbated by the local lung damage in this study. Improving respiratory health may be one of the essential ways to prevent PM-induced dyslipidemia in broilers. More studies are necessary to further reveal the mechanism of lung injury and abnormal liver lipid metabolism in broilers induced by PM from poultry houses. It will provide an effective target for preventing and controlling respiratory diseases and liver damage in broilers induced by PM in broiler houses.

## Figures and Tables

**Table 1:** Effects of particulate matter exposure on growth performance of broilers.

Items	Control	4 mg·m^−3^	8 mg·m^−3^
E7			
Average daily gain (g/day)	56.71 ± 2.73 ^A^	50.12 ± 2.21 ^AB^	44.35 ± 2.16 ^B^
Average daily feed intake (g/day)	95.53	97.76	90.75
Feed/Gain	1.68	2	1.85
E14			
Average daily gain (g/day)	67.52 ± 3.98	73.81 ± 3.04	65.05 ± 5.66
Average daily feed intake (g/day)	124.29	122.95	109.91
Feed/Gain	1.84	1.73	1.85
R7			
Average daily gain (g/day)	81.43 ± 5.34	92.14 ± 3.83	83.33 ± 4.56
Average daily feed intake (g/day)	264.29	279.52	275.37
Feed/Gain	2.78	2.60	2.94

Values of average daily gain represent the mean ± SEM of the group (n = 8). Different capital letters indicate significant differences between groups (*p* < 0.01); the same letters indicate no difference between groups (*p* > 0.05). E7, exposure for 7 days; E14, exposure for 14 days; R7, recovery for 7 days.

## Data Availability

All data in this article are presented in the article, and the original data are available from the corresponding author upon reasonable request.

## Supplementary Materials

The following supporting information [50] can be downloaded at: https://www.mdpi.com/article/10.3390/metabo13030363/s1, Table S1: The actual exposure concentrations of particulate matter (mg·m^−3^) with different particle sizes in each group during periods of exposure and recovery; Table S2: Sequences and parameters of gene primers used for quantitative real-time PCR; Table S3: Effects of particulate matter exposure on organ indices of broilers; Figure S1: Real-time monitoring of actual concentrations of PM_2.5_ (A), PM_10_ (B), and TSP (C) during exposure and recovery in chambers of control and PM-exposed groups. E, exposure; R, recovery.
