# Supplementary material for: Exposure to Particulate Matter in the Broiler House Causes Dyslipidemia and Exacerbates It by Damaging Lung Tissue in Broilers
Source: Metabolites. 2023 Feb 28;13(3):363. doi: 10.3390/metabo13030363 (PMC10052321; doi:10.3390/metabo13030363)
Supplement: Supplementary file 1 [file metabolites-13-00363-s001.zip › metabolites-2237170-supplementary.pdf]

Exposure to particulate matter in the broiler house causes dyslipidemia through damaging lung tissue in broilers

Dan Shen[1], Qi Guo[1], Kai Huang[1], Weijia Mao[1], Kai Wang[1], Wenjie Zeng[1], Yansen Li[1], Zhendong Guo[2], Kentaro Nagaoka [3], Chunmei Li[1,*]

[1] Research Centre for Livestock Environmental Control and Smart Production, College of Animal Science and Technology, Nanjing Agricultural University, Nanjing 210095, China

[2] Military Veterinary Research Institute, Academy of Military Medical Sciences, Changchun 130117, China.

[3] Laboratory of Veterinary Physiology, Department of Veterinary Medicine, Faculty of Agriculture, Tokyo University of Agriculture and Technology, Tokyo, 183-8509, Japan

[*] Corresponding author: Professor Chunmei Li

E-mail: chunmeili@njau.edu.cn, Tel.: 00 86 25 84395971

National Natural Science Foundation of China (No. 32072781), China Postdoctoral Science Foundation (2022M721651), Jiangsu Agricultural Industry Technology System (JATS(2022)480).

Table S1 The actual exposure concentrations of particulate matter ($mg \cdot m^{-3}$) with different particle sizes in each group during period of exposure and recover.

Items	Broiler house[1]	Experiment chambers (Exposure)			Recover
		Control	4 $mg \cdot m^{-3}$	8 $mg \cdot m^{-3}$	
PM_1	0.571 ± 0.017	0.254 ± 0.052	1.157 ± 0.126	1.710 ± 0.099	0.419 ± 0.009
$PM_{2.5}$	0.713 ± 0.021	0.256 ± 0.052	1.264 ± 0.120	1.976 ± 0.083	0.421 ± 0.009
PM_4	0.991 ± 0.029	0.260 ± 0.052	1.402 ± 0.115	2.343 ± 0.065	0.427 ± 0.010
PM_{10}	2.499 ± 0.077	0.327 ± 0.052	2.344 ± 0.106	4.479 ± 0.097	0.536 ± 0.019
TSP	4.033 ± 0.129	0.657 ± 0.117	4.359 ± 0.078	8.111 ± 0.130	1.198 ± 0.077

[1] The data cited from a published paper [50].

Table S2 Sequences and parameters of gene primers used for quantitative real-time PCR.

Gene	Accession No.	Primer sequences	Primer size, bp
Fas	NM_205155.3	F: 5’ GCTAAGATGGCATTGCACGG 3’	20
		R: 5’ TCCATTCAGTTCCAGACGGC 3’	20
Hmgcr	XM_015277227.2	F: 5’ CAAATGCGGTTTCCTGTCCTT 3’	21
		R: 5’ AGTGGCTACACACCTCTTCC 3’	20
Hmgcs2	XM_422225.6	F: 5’ GCTCTGTGCATCCCAAAACG 3’	20
		R: 5’ CCTTATCAGCACATCGGGCA 3’	20
PPARα	XM_015289937.2	F: 5’ CTCTGCCCTTGACGGAAAGT 3’	20
		R: 5’ AAGGTTGAAACAGAAGCCGC 3’	20
β-actin	NM_205518.1	F: 5’ TTGTCCACCGCAAATGCTTC 3’	20
		R: 5’ AAGCCATGCCAATCTCGTCT 3’	20

Table S3 Effects of particulate matter exposure on organ indices of broilers.

Items	Control	4 mg·m^{-3}	8 mg·m^{-3}
**E 7**			
Heart (g)	5.91 ± 0.588	5.86 ± 0.331	6.32 ± 0.619
Heart index (%)	0.66 ± 0.039	0.67 ± 0.048	0.76 ± 0.073
Lung (g)	4.93 ± 0.324	5.56 ± 0.781	5.25 ± 0.583
Lung index (%)	0.56 ± 0.040	0.61 ± 0.060	0.626 ± 0.053
Liver (g)	24.81 ± 1.758	24.79 ± 1.681	21.31 ± 1.458
Liver index (%)	2.87 ± 0.345	2.76 ± 0.085	2.58 ± 0.166
Spleen (g)	1.15 ± 0.136	1.54 ± 0.160	1.19 ± 0.103
Spleen index (%)	0.13 ± 0.010	0.17 ± 0.016	0.14 ± 0.005
**E 14**			
Heart (g)	8.56 ± 0.413	8.17 ± 0.431	8.61 ± 0.498
Heart index (%)	0.64 ± 0.045	0.63 ± 0.068	0.72 ± 0.043
Lung (g)	7.73 ± 0.512	7.41 ± 0.677	6.59 ± 0.356
Lung index (%)	0.57 ± 0.036	0.54 ± 0.023	0.55 ± 0.045
Liver (g)	34.28 ± 1.446	33.90 ± 2.407	40.58 ± 3.48
Liver index (%)	2.55 ± 0.164	2.50 ± 0.076	3.58 ± 0.612
Spleen (g)	1.91 ± 0.203	1.93 ± 0.174	2.45 ± 0.644
Spleen index (%)	0.14 ± 0.010	0.14 ± 0.008	0.22 ± 0.077
**R 7**			
Heart (g)	10.22 ± 0.734	10.26 ± 0.574	9.73 ± 0.975
Heart index (%)	0.53 ± 0.029	0.53 ± 0.042	0.53 ± 0.050
Lung (g)	9.71 ± 0.711	10.35 ± 1.265	8.69 ± 1.157
Lung index (%)	0.51 ± 0.035	0.53 ± 0.063	0.48 ± 0.070
Liver (g)	41.92 ± 2.035	43.52 ± 2.101	47.72 ± 3.230
Liver index (%)	2.21 ± 0.140	2.25 ± 0.137	2.61 ± 0.171
Spleen (g)	2.45 ± 0.265	3.27 ± 0.693	3.15 ± 0.399
Spleen index (%)	0.13 ± 0.013	0.17 ± 0.034	0.17 ± 0.023

Each value represents the mean ± SEM of the group (n = 8).

E7, exposure for 7 days; E14, exposure for 14 days; R7, recover for 7 days.

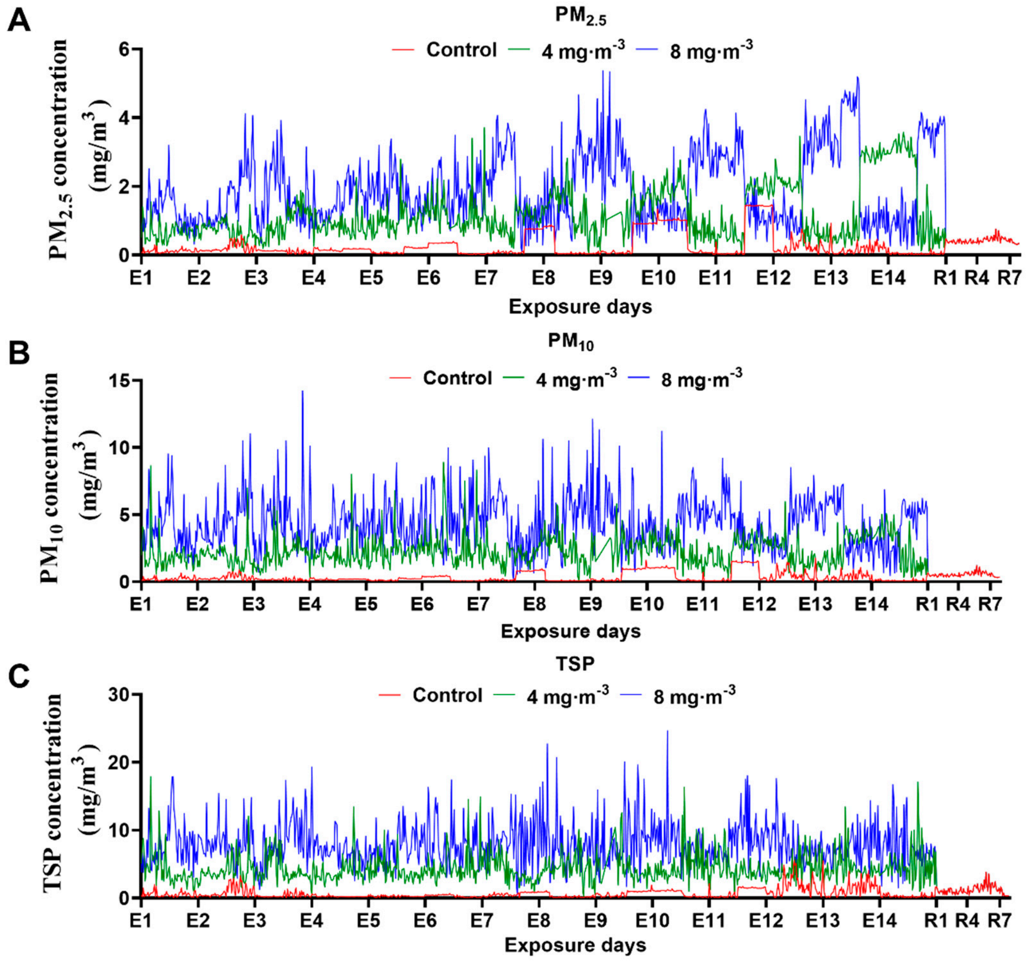

Figure S1. Real-time monitoring of actual concentrations of $PM_{2.5}$ (A), PM_{10} (B) and TSP (C) during exposure and recover in chambers of control and PM-exposed groups. E, exposure; R, recover.

References

50. Shen, D.; Guo, Z.; Huang, K.; Dai, P.; Jin, X.; Li, Y.; Li, C. Inflammation-Associated Pulmonary Microbiome and Metabolome Changes in Broilers Exposed to Particulate Matter in Broiler Houses. *J. Hazard. Mater.* **2022**, *421*, 126710. https://doi.org/10.1016/j.jhazmat.2021.126710.
